# Factors influencing health care workers’ implementation of tuberculosis contact tracing in Kweneng, Botswana

**DOI:** 10.11604/pamj.2016.24.229.7004

**Published:** 2016-07-13

**Authors:** Lebapotswe Tlale, Rosemary Frasso, Onalenna Kgosiesele, Mpho Selemogo, Quirk Mothei, Dereje Habte, Andrew Steenhoff

**Affiliations:** 1 University of Botswana School of Medicine, Gaborone, Botswana; 2 Perelman School of Medicine, University of Pennsylvania , Philadelphia, USA; 3 Masters of Public Health Program, School of Medicine and School of Social Policy & Practice, Philadelphia, USA; 4 Kweneng East District Health Management Team TB Unit, Molepolole, Botswana; 5 Botswana Accountancy College, Gaborone, Botswana; 6 Botswana-UPenn Partnership, Gaborone, Botswana; 7 Children’s Hospital of Philadelphia, Philadelphia, PA, USA

**Keywords:** Tuberculosis, policy, guidelines

## Abstract

**Introduction:**

TB contact tracing rates remain low in high burden settings and reasons for this are not well known. We describe factors that influence health care workers' (HCW) implementation of TB contact tracing (CT) in a high TB burden district of Botswana.

**Methods:**

Data were collected using questionnaires and in-depth interviews in 31 of the 52 health facilities in Kweneng East Health District. Responses were summarized using summary statistics and comparisons between HCW groups were done using parametric or non-parametric tests as per normality of the data distribution.

**Results:**

One hundred and four HCWs completed questionnaires. Factors that influenced HCW TB contact tracing were their knowledge, attitudes and practices as well as personal factors including decreased motivation and lack of commitment. Patient factors included living further away from the clinic, unknown residential address and high rates of migration and mobility. Administrative factors included staff shortages, lack of transport, poor reporting of TB cases and poor medical infrastructure e.g. suboptimal laboratory services. A national HCW strike and a restructuring of the health system emerged as additional factors during in-depth interviews of TB coordinators.

**Conclusion:**

Multiple factors lead to poor TB contact tracing in this district. Interventions to increase TB contact tracing will be informed by these findings.

## Introduction

Contact tracing (CT) contributes to improving early case detection of tuberculosis (TB). A recent study from Botswana reported a new TB diagnosis yield of 2.2% from CT of household contacts of pediatric TB cases [1]. This is very similar to the pooled yield of 2.3% described in a meta-analysis of 23 adult TB case-focused studies [2]. This was a systematic review and meta-analysis of all studies worldwide reporting the prevalence of TB and latent TB infection, and the annual incidence of TB among contacts with TB. Despite clear CT recommendations in both international and national guidelines, implementation of TB CT in lower-income, high TB burden country remains limited [3].

A key step towards improving CT is to understand why implementation has been limited. In high burden TB settings the factors that adversely influence implementation of CT are poorly described. In low burden TB settings, the literature describes some barriers to CT. In the United States, a review of policies and structured interviews of TB program managers and staff described communication, structural and patient level barriers as challenges when conducting contact investigations [4]. A systematic review of factors influencing the implementation of clinical guidelines for health care professionals described that guideline complexity, implementation strategies, characteristics of professionals and patient factors all influenced implementation of clinical guidelines [5]. It is likely that many of these factors play a role in the implementation of CT in high burden TB settings.

Although previous studies have evaluated aspects of guideline implementation [5], other aspects that are likely crucial in developing country settings have not been adequately explored. These include administrative, personal and patient factors as well as knowledge, attitudes and practices of health workers. Botswana is both a high burden TB and HIV setting - 2011 annual TB notification rate was 505 per 100,000 population and population HIV seroprevalence was 17.6% [6]. The CT target set by the Botswana National TB Program since 2011 is 80%. Although national rates of TB CT are not routinely reported, personal communication with TB coordinators' suggests that this is successfully completed in less than 25% of adult smear positive cases [7]. Our objective was to quantitatively determine and qualitatively describe the factors that influence HCW implementation of TB CT in this high TB-HIV prevalent setting. Analysis of these data will inform interventions to improve rates of CT.

## Methods

### Setting

Botswana has a government funded public health system. Kweneng East Health District is adjacent to Gaborone, the nation's capital, and in 2012 had a high TB notification rate of 494 per 100 000 population and an HIV seroprevalence among adults of 19% [7]. The district is home to 217829 people which is 11% of the country's population and covers an area of 35 890 km^2^. It contains urban, semi-urban and rural settings. There are three hospitals and 49 clinics and health posts in the district [7]. In Botswana contact tracing is generally done by nurses and health education assistants [8]. When a new case of PTB is diagnosed by a medical officer or nurse, the TB focal person at the facility or the community TB care volunteer must identify all close contacts and screen them for TB symptoms [6]. While TB is taught in a general fashion in all HCW curricula in Botswana, none of the HCW groups receive intense training on CT as part of their tertiary education. Once HCW are deployed into the health system, contact tracing training is usually facilitated by the infection control nurse and the District TB coordinator and all the health workers are expected to attend at least one training.

### Study design and population

A mixed method approach was employed. In phase one of the project we used a self-administered questionnaire to elicit factors that influence HCW's implementation of CT. In addition to demographic information, the questionnaire contained 27 closed-ended questions and 6 open-ended questions which focused on various aspects of TB contact tracing. The target group was all HCWs working with TB patients: those at clinics, health posts, the primary hospital or the TB ward at the district hospital. The HCWs were nurses, medical doctors, health education assistants and TB focal persons which included two TB coordinators. Each facility has a TB focal person and there are two TB coordinators in Kweneng East District. In phase two we conducted in-depth interviews with the two district TB coordinators. TB coordinators focus on all aspects of TB prevention, control and treatment. They are the key link between each district in Botswana and the National TB Program. Kweneng East District has two TB coordinators, which is more than any other District, and this is because the district has the highest number of TB cases in the country. These interviews allowed the TB coordinators to express their in-depth opinions regarding factors that influence HCW implementation of CT. Each TB coordinator was formally interviewed by one of the authors (Lebapotswe Bahumi Tlale-LBT) using a semi-structured interview guide. The interview guide was designed by the study team (LBT and his two academic supervisors - Dereje Habte and Andrew Pierre Steenhoff - APS) to elicit the TB coordinators' attitudes, opinions, feelings and behaviors. Participant responses were hand recorded by the researcher (LBT) in a study notebook. The researcher-interviewer validated captured information by repeating the questions to ensure accuracy of the notes taken. Each interview lasted for about 45 minutes. A code book was created and then the data were independently reviewed and coded by two investigators (LBT, APS). Coding differences were resolved by consensus. We report the major themes that emerged.

### Sampling technique and sample size

For the quantitative part a non-probability sampling technique was employed. Study participants were recruited from 18^th^ January 2013 until 29^th^ May 2013. We estimated 160 potentially eligible subjects in the district. With an estimated 20% declination rate, the overall potential study cohort was 128 subjects. We anticipated that there would be a saturation of themes at or before 100 subjects and hence the target sample size was 100 participants [9, 10]. For the qualitative phase, we planned to interview both of the district's two TB coordinators.

### Definitions

**Health education assistant**: a health worker with a minimum of primary school certificate and 6 months of health training, who is responsible for education and care of the community.

**Medical officer**: a medical doctor who has completed a general internship but has no specialty training.

**TB focal person**: a health worker, either a nurse or a health education assistant, who is responsible for TB activities in the village and or district. TB coordinators were included in this group.

**Nurse:** a health worker with a minimum of a 3 year diploma in nursing. Assessment of work place motivation was defined by evaluating all of the following factors: monthly income, distance from work, and job satisfaction.

**Outcomes** The primary outcomes of interest were factors that influence HCWs implementation of CT and TB coordinators' perceptions of factors that hinder implementation of CT. Additionally, we examined the level of acceptable CT knowledge, defined as the HCW's ability to list all the steps required to conduct CT and knowing which forms are used. Motivation was measured on how HCWs described their obligation to conduct CT. The administrative factors as reported by HCWs included those controlled by the health management and that were out of the sphere of influence of both HCWs and their patients. The secondary outcomes were the recommendations that HCWs volunteered regarding how CT could be improved in the district.

### Analysis

Interview notes were transcribed verbatim and uploaded into Microsoft excel to facilitate coding. We employed common coding techniques to identify themes in the responses to all open-ended questions. Themes identified in the analysis of transcribed notes from the interviews with TB coordinators (n=2) were compared to those identified in the analysis of the responses to the open ended questions asked to HCWs (n=104). This provided an opportunity to examine two perspectives on the experience of interest. Responses to close-ended questions were summarized using summary statistics. Responses of different HCW groups were compared using parametric or non-parametric tests as per normality of the data distribution. Nurses were chosen as the reference group when analyzing by HCW. Nurses were chosen as they are the core HCW group in Botswana and each clinic has at least one nurse. To compare proportions between groups, chi square test was used. A test of proportions was used to determine which factors were significant. A P value less than 0.05 was deemed statistically significant. When numbers were too small a Fisher exact test was used. Data were analyzed using STATA version 12 (College Station, TX).

### Ethics

Each participant signed an informed consent. Approval was given by the Institutional Review Boards of Botswana's Ministry of Health, the University of Botswana and Kweneng East District Health Management Team.

## Results

### Study participants and sites

**Figure 1:**
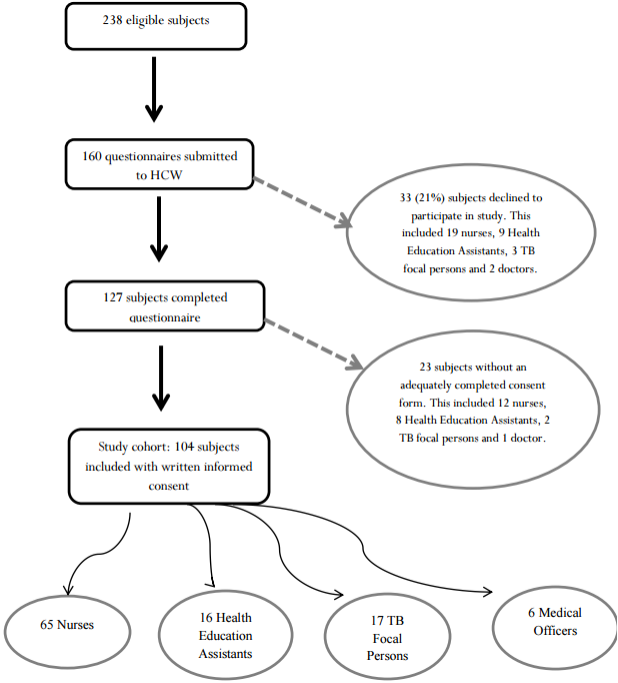
Flow diagram of study enrollment

**Figure 2:**
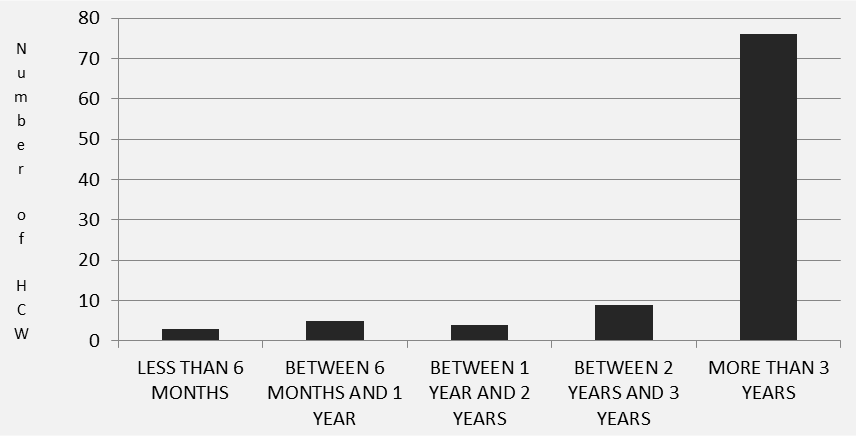
Experience of HCW working with TB patients

A total of 160 questionnaires were disseminated to HCWs around Kweneng East Health District. Figure 1 summarizes study enrollment yielding a total sample size of 104 HCWs. Thirty three (21%) HCWs declined to participate in the study, stating that they had no time to fill in the questionnaire. Twenty three health care workers did not complete the consent form and were not included in the analysis. Figure 2 illustrates HCW level of experience working with TB patients. The relative proportions of HCW groups in the study reflected that in the district overall (Table 1). Participating HCWs worked at 31 (60%) of 52 health facilities in the district. In discussions with the District TB coordinators, the coordinators commented that the TB contact tracing rate was similarly low (around 25%) in the 31 facilities sampled in this study as compared to the 21 facilities that were not sampled[7]. This value of 25% was calculated by the TB coordinators who, although there is no national approach to assessing contact tracing in Botswana, gathered data on 2011 Kweneng District TB cases, their number of contacts and the proportion of these contacts who had contact tracing done [7]. Each HCW completed a data collection form. Five forms missed the name of the health facility site, but they were included in the final analysis of the paper. The study health facilities were located in 11 (46%) of 24 villages and all 31 were located in rural areas. In one form, the participant did not disclose the village of origin and this form was also included in the analysis.

**Table 1:** Profession of subjects who participated in the study as compared to the health care work force in the district overall

Occupation	No. in study (% of total in study)	No. in Kweneng East district (% of HCW in district)	Proportion of cadre sampled in the study (study n/total N in the district)	Comparison of proportion of those in study vs total in the district *P* value
Nurses	65 (63%)	145 (61%)	45%	0.78
Health Education Assistants	16 (15%)	41 (17%)	39%	0.67
TB focal persons	17 (16%)	37 (15%)	46%	0.85
Medical officers	6 (6%)	15 (6%)	40%	0.85
Total	104 (100%)	238 (100%)	44%	-

### Quantitative findings

When comparing HCW cadres (health education assistants, TB focal persons and doctors) for knowledge, there was a wide range (17-69%) of HCWs with acceptable knowledge but the knowledge levels did not differ by group when compared to nurses (Table 2). With regard to training in CT, health education assistants reported the highest proportion (93%) of training as compared with other groups (50 to 60%). The majority (100%) of HCW assessed TB CT as a “good” (33%) or “very good” (67%) tool to identify new TB cases (Table 3). Frequency of conducting TB CT varied by group with health education assistants most frequent and medical officers least frequent. The majority of HCW answered that their health care facility was not achieving the national CT target. The majority of HCW responded that they did not think that distance from work, dissatisfaction with the job, excessive patient numbers, unavailability of supplies like N95 masks, poor policy and guidelines and poor reporting and recording were factors that contributed to low TB CT rates in the district (Table 4). These responses were consistent across HCW groups. With regards to administrative factors shortage of stuff and transport were the most prominent (Table 5).

**Table 2:** Knowledge of and training in TB contact tracing by occupation

Occupation	Proportion working with acceptable knowledge of TB CT*	Knowledge: comparison between this group and nurses *p* value	Proportion trained to do TB contact tracing #	Training: comparison between this group and nurses *p* value
Nurses	37/64 (58%)	Reference	35/65 (54%)	Reference
Health Education Assistants	11/16 (69%)	0.42	14/15 (93%)	0.005
TB focal person and others##	9/17 (53%)	0.71	10/17 (59%)	0.71
Medical Officers	1/6 (17%)	0.06	3/6 (50%)	0.86

* Acceptable knowledge defined as knowing both the steps to complete contact tracing as well as knowing about Botswana’s National TB contact tracing forms.

# Defined as having attended at least one training session on how to do contact tracing.

## This included 2 TB coordinators, 2 health education technicians, 4 health education assistants and 9 nurses.

**Table 3:** Practices of health care workers when conducting tuberculosis contact tracing

		Nurse	Health Education Assistant	TB focal person and others	Medical officer	Total
**How would you rate TB contact tracing as an important tool to identify new cases?**	Very good	76%	50%	40%	100%	67%
	Good	20%	50%	60%	0%	33%
	Fair	2%	0%	0%	0%	1%
	Poor	2%	0%	0%	0%	1%
	Very poor	0%	0%	0%	0%	0%
**How often do you conduct TB contact tracing?**	Never	3%	6%	80%	0%	22%
	Less than 1/year	30%	19%	20%	100%	42%
	About 1/month	37%	13%	0%	0%	13%
	About 1/week	30%	38%	0%	0%	17%
	More than 1/week	0%	25%	0%	0%	6%
**Do you think that your health facility is achieving the national TB contact tracing target?**	Yes	13%	31%	0%	0%	11%
	No	54%	46%	80%	100%	70%
	Not sure	33%	23%	20%	0%	19%

**TB:** tuberculosis; **CT:** contact tracing

**Table 4:** Personal and patient factors influencing health worker implementation of TB contact tracing

	Proportion who said “Yes” (%)					
Factors that influence my implementation of contact tracing	Nurses	Health Education Assistants	TB focal persons and others	Medical officers	Total	P value comparing proportion who said “yes” vs “no” for each factor
Commitment of other HCW toward CT	40/54 (74%)	6/9 (67%)	8/9 (89%)	0/4 (0%)	54/76 (71%)	*P*=0.003
My monthly income	13/55 (24%)	5/10 (50%)	4/12 (33%)	0/4 (0%)	22/81 (27%)	*P*=0.0096
My distance from work	24/51 (47%)	6/8 (75%)	7/11 (64%)	2/4 (50%)	39/74 (53%)	*P*=0.72
Involvement of other HCW	49/58 (84%)	7/9 (78%)	7/9 (78%)	2/4 (50%)	65/80 (81%)	*P*=0.0000
Dissatisfaction with my job	23/50 (46%)	6/8 (75%)	7/11 (64%)	2/4 (50%)	38/73 (52%)	*P*=0.79
Patient numbers too many	25/53 (47%)	5/9 (56%)	7/11 (64%)	1/2 (50%)	38/75 (51%)	*P*=0.93
Distant location of patient	48/56 (86%)	11/11 (100%)	14/14 (100%)	3/4 (75%)	76/85 (89%)	*P*=0.0000
Migration and mobility of patients	59/61 (97%)	11/11 (100%)	11/12 (92%)	5/5 (100%)	86/89 (97%)	*P*=0.0000
Unknown patient address	41/52 (79%)	5/7 (71%)	13/13 (100%)	3/4 (75%)	62/76 (82%)	*P*=0.0000

NOTE: 1. Yes refers to those who responded by saying that they strongly agree and agree both combined e.g. Yes SA + A 2. No refers to those who responded by saying that they strongly disagree and disagree both combined e.g. No = SD + D 3. Those who answered “fairly” were not included in the analysis 4. Those who did not respond to the question were not included in the analysis

**Table 5:** Administrative factors influencing health worker implementation of TB contact tracing

	Proportion who said “Yes” (%)					
Factors that influence my implementation of contact tracing	Nurses	Health Education Assistants	TB focal persons and others	Medical officers	Total	Comparing proportion who said “yes” vs “no” for each factor
Shortage of staff	60/62 (97%)	9/9 (100%)	13/13 (100%)	3/4 (75%)	85/88 (97%)	*P*=0.0000
Lack of transport	61/62 (98%)	9/10 (90%)	13/13 (100%)	5/5 (100%)	88/90 (98%)	*P*=0.0000
Lack of medical infrastructure	42/53 (79%)	0/6 (0%)	9/12 (75%)	2/5 (40%)	53/76 (53%)	*P*=0.008
Unavailability of supplies	25/46 (54%)	3/8 (38%)	5/11 (45%)	2/5 (40%)	35/70 (50%)	*P*=1.00
Poor reporting and recording of TB contact tracing	35/52 (67%)	9/11 (82%)	9/11 (82%)	4/4 (100%)	57/78 (73%)	*P*=0.003
Poor policy and guidelines	18/48 (38%)	2/6 (33%)	8/12 (67%)	1/4 (25%)	29/70 (41%)	*P*=0.47

### Qualitative findings

Analysis of data obtained from the in-depth interviews with TB coordinators highlighted that several factors contributed to lower rates of TB CT including those noted above. Additionally, two unique themes emerged that had not been identified in the quantitative analysis of data collected from the other 104 HCWs. The first theme, ***Fallout from 2010 Health System Transition***, summarized the coordinators experiences following the 2010 change in the structure of Botswana's National Health System. During this change clinics and District Health Teams which had previously been managed by the Local Government Authority were moved under the leadership of the Ministry Of Health. When asked how the transition might have contributed to poor TB CT rates, the interviewees responded that the transition process was not smooth. The result was that various aspects of health delivery were somewhat compromised. In addition, participants reported that resources (human resources, infrastructure and transport) that had previously been reserved for the hospitals were, after the transition, under additional stress as these resources were now assigned to serve both hospitals and clinics. “One of the major problems we face with contact tracing is that there is a lack of transport. The transport situation used to be better before the decentralization of clinic from Local Government to Ministry of Health. This was not a smooth process - even currently some of the health workers were asked to leave their houses because they (the houses) belong to Local Government (*and not to the Central Government*)”. District TB coordinator. The second theme, ***Fallout from 2011 Public Servants Strike***, summarized the TB coordinators" experiences during the 2011 national HCW strike, during which some HCWs were fired. This led to new HCWs being employed or transferred into the region. This included HCWs from other countries who were unfamiliar with the Botswana health system. “During and after the strike in 2011, health workers were fired and new ones were hired. The majority of these health workers was not familiar with the system and also had to be trained on many aspects of TB. Some of them were foreigners and it was a problem for them to move around the district.” Hospital TB coordinator.

## Discussion

This study describes that multiple factors play a role in low TB CT rates in a high TB-HIV prevalent health district of Botswana. For HCW, these included their knowledge, attitudes and practices as well as decreased motivation and lack of commitment. Patient factors included living further away from the clinic, unknown residential addresses and high rates of migration and mobility. Administrative factors included staff shortages, lack of transport, poor reporting of TB cases and poor medical infrastructure. A restructuring of the health system and a national HCW strike, in 2010 and 2011 respectively, emerged as additional factors.

The HCW related factors included suboptimal knowledge, attitudes and practices. For knowledge, poor levels of TB CT training contributed to this poor knowledge with fewer than 60 percent of all groups apart from health education assistants having been trained. This is a similar finding to White et al. who reported that only 40% of Jamaican health care workers demonstrated sufficient knowledge of TB [11]. This knowledge gap is an important factor that likely affects guideline implementation [11]. Additionally even among cadres with a reasonable level of contact tracing training, including the health education assistants and doctors, their knowledge was still unacceptably low. This could be due to a number of factors including suboptimal quality or technique of training or that knowledge retention may be insufficient. This is an area that needs further investigation.

HCW attitudes were interesting. Nearly all HCWs thought that TB CT was an important tool to identify secondary TB cases. This is encouraging as HCW clearly understood the importance of TB CT. However, only 42 percent of HCW responded that they were aware of the national guidelines. Despite this more than a third of all HCW reported that poor policies and guidelines were a contributing factor. This again highlighted the need for optimal guideline implementation including effective dissemination and buy-in by HCW. HCW motivation has been shown to be important with more motivated HCW's delivering better care [11]. Overall HCW were satisfied. Khanlub described that the main factors that correlated with overall job satisfaction in health center-based HCW in Laos PDR were effective conflict resolution, good relationships with co-workers, and clear organizational structure [12]. Although we postulated that poor financial reimbursement might be a demotivating factor, this was not the case. Although not formally assessed, this may be because HCW in Botswana feel that they are fairly reimbursed. This differed from the Laos PDR study probably reflecting that Botswana HCW are better paid than those in Laos PDR. HCW also responded that distance from work was not a contributing factor possibly because most live near to their work which is facilitated by the provision of housing by the Botswana Government to health workers. Although not assessed in this study, many health education assistants in Botswana are people who have grown up in the area they work and their local knowledge may also mitigate transport challenges.

Patient factors can also be barriers to CT [5]. In our study the patient factors that were barriers to CT included patient migration, distant location, mobility and unknown addresses. Jesus et al. described that patient migration in Barcelona lead to low CT rates [13]. People in Botswana frequently have two or more homes in different places due to working in a city or town and then having a farm in a more rural location. This explains some of the challenges with TB CT including HCW concern regarding patients living in a distant location, their mobility and unknown addresses. Large patient numbers were not noted to be a factor by HCWs. This is in keeping with the adequate patient to health worker ratio reported for Botswana [12, 13]. Administrative factors affect the functioning of a health system. The most outstanding factors included shortage of staff and transport. The reported shortage of staff apparently contradicts the finding that patient numbers were not excessive - however this may reflect an uneven distribution of HCW across Botswana, particularly between urban and rural sites. A similar challenge has been reported from South Africa where there is an unequal distribution of HCW between the private and public sectors and between urban and rural areas [14]. For transport, Botswana's Assistant Minister of Health has reported that only 42% of allocated vehicles are available for use [15]. Also as highlighted by the TB coordinators, many transport resources that belonged to clinics were surrendered to the Ministry of Local Government. Other administrative factors were a lack of medical infrastructure and poor recording and reporting of TB CT. Similarly, in Uganda, a lack of laboratory diagnostics, medical infrastructure and medical supplies were barriers to provision of integrated TB/HIV care [16]. For poor reporting and recording, this may reflect the use of manual TB registers in Botswana. These are often incomplete as reported in a recent review [17]. The other problem in Botswana is that, although there is a standard TB CT form which is implemented at clinic level, there is currently no formalized central reporting of CT rates to the National TB program.

Although our study design may have some limitations, the design affords the opportunity to generate hypotheses for future analytical and experimental studies. The triangulation approach enabled finding of agreement and validation of results through two research methods. Our study was confined to one district in Botswana and hence, due to many inter-district differences, our findings cannot be generalized to the whole country. Only 62% percent of the health facilities and 44% of the HCW were sampled in the survey. This could lead to some selection bias given that convenience sampling was used to approach potential subjects and that rural clinics were sampled. However, the district TB report showed that there was no difference in contact tracing rates between the 40% of the unsampled facilities as compared to those sampled in the study [7]. The other potential limitation is incomplete sampling of the study population as 18% of potential study participants were excluded due to incomplete consent forms - these subjects were similar by health cadre to those enrolled (Figure 1).

However, as they did not have a completed consent form, we did not assess anything further about them. There was likely minimal recall bias as the questions in the questionnaire rarely asked about recalling distant events. Additionally about half (53%) of TB focal persons were nurses and this may confer a risk for confounding by indication. Twenty one percent of HCW approached declined to participate. These groups were not significantly different by health cadre but, as they declined to participate, we did not assess anything further about them. We have no reason to suspect that this group was significantly different to study participants but if they were then this would introduce the risk of potential sampling bias. The other limitation is that the qualitative data was based on only two interviews. When taking notes a lot of effort is put into writing down than what is being said, this could lead to some of the vital information being missed, but one of the advantages of using this technique is that notes are easy to look through at the end of the interview when you want to make certain that you didn´t miss anything [17].

## Conclusion

In conclusion, despite health care workers knowing the importance of TB CT for the prevention and control of TB we describe personal, patient and administrative factors that were barriers to achieving optimal CT targets. However, the extent to which any individual factor played a role was not measured in this descriptive paper. This report provides data for hypothesis generation for future analytical and experimental studies. Multi-pronged interventions need to be devised to improve the situation.

### What is known about this topic

- Several studies have assessed the knowledge, attitudes and practices of health workers and patients on TB in general;
- Also several studies have assessed adherence to clinical guidelines in general.

### What this study adds

- Few studies have assessed the knowledge, attitudes and practices of health care workers towards TB contact tracing;
- Our study will focus on this elements and how they affect implementation of contact tracing in a high burden HIV/TB setting;
- No or very few specific studies have assessed the adherence of contact tracing guidelines. This study describes how health workers view the importance TB/CT guidelines.
